# Exercise intensity and shooting position modulate fixation behavior and 2-point shooting accuracy in elite female basketball players: An eye-tracking study

**DOI:** 10.1371/journal.pone.0348017

**Published:** 2026-04-30

**Authors:** Xiaokun Zhang, Sunnan Li, Chunzhou Zhao

**Affiliations:** 1 College of Physical Education Science, Harbin Normal University, Harbin, China; 2 College of P. E and Sports, Beijing Normal University, Beijing, China; 3 College of Physical Education, Chizhou University, Chizhou, China; Universiti Malaysia Terengganu, MALAYSIA

## Abstract

Visual attention plays a crucial role in basketball shooting, yet how it adapts under varying exercise intensities and from different shooting positions remains poorly understood, especially in elite female athletes. This study examined the effects of exercise intensity (low, moderate, high) and shooting position (left 45°, 90°, right 45°) on fixation behavior and 2‑point shooting accuracy in elite female basketball players. Twenty‑two players from a championship‑winning university team performed two‑point shots from three positions under three intensity conditions, each defined by heart‑rate zones (%HRmax). Fixation metrics (number of fixations, fixation duration, distribution) were recorded using Tobii Glasses 3. Data were analyzed using two‑way repeated‑measures ANOVA and generalized linear mixed‑effects models (GLMMs) with a binomial distribution for trial‑level accuracy; Pearson correlations are reported descriptively. Exercise intensity significantly influenced all fixation metrics. High intensity led to increased fixations across all areas of interest (hoop, backboard, net) and longer total fixation duration, indicating higher cognitive load. Moderate intensity was associated with the lowest total number of fixations and shortest duration, reflecting efficient visual processing. Shooting position also affected fixation: the 90° position attracted the most fixations and longest duration on the hoop (*F* = 4.56, *p* = 0.017, ηp2 = 0.19, 95% CI [0.02, 0.37]), while the 45° positions shifted attention toward the backboard. Fixation duration on the hoop positively correlated with accuracy under high intensity (*r* = 0.499, *p* = 0.021, 95% CI [0.10, 0.76]), whereas number of fixations negatively correlated with accuracy across intensities. Moderate intensity promotes optimal visual‑attentional control and shooting accuracy in elite female athletes, whereas high intensity disrupts fixation stability and increases cognitive load. Position‑specific adaptations in visual strategy were also observed. These findings support the use of intensity‑ and position‑based visual training to enhance shooting performance under realistic game conditions.

## Introduction

Shooting accuracy is a critical determinant of success in basketball, heavily reliant on athletes’ visual attentional control [1]. Recent research in sports cognition has revealed systematic differences in visual search strategies between elite and novice athletes, manifested in fixation allocation, saccade trajectory stability, and information extraction efficiency [2]. Of particular significance is the Quiet Eye (QE) period—defined as the final fixation duration on a critical target (e.g., the hoop) prior to movement execution—which has been established as a key predictor of shooting accuracy [3,4]. However, most existing studies have been confined to single shooting positions (e.g., the free‑throw line) or static laboratory conditions [5], failing to adequately simulate the dynamic visuomotor adaptation mechanisms required in real‑game scenarios involving multiple positions and varying intensity levels [6].

The FISU World University Games, the world’s premier university sports event, feature elite athletes who demonstrate exceptional technical and tactical proficiency, offering an ideal sample for investigating high‑level visuomotor coordination [7]. Although several studies have explored visual attention characteristics in basketball players, research focusing on female elite athletes—particularly under varied exercise intensities and across different shooting positions—remains scarce [8–10]. Moreover, prior work has predominantly centered on free throws [11,12], with less attention paid to two‑point field goals, which are common in open play [6].

The advent of wearable eye‑trackers (e.g., Tobii Glasses) has enabled researchers to collect data in natural training environments with higher ecological validity [13]. These devices allow synchronous recording of multiple oculometric measures, such as number of fixations, fixation duration, total number of fixations, total fixation duration, and fixation distribution, providing a rich dataset for in‑depth analysis of visual attention patterns [3,14].

Guided by Dynamic Systems Theory (which emphasizes how behavior emerges from the interaction of multiple constraints) and Attentional Control Theory (which describes how cognitive control shifts under stress or high demand), this study aims to systematically investigate: (1) how exercise intensity (low, moderate, high) and shooting position (left 45°, 90°, right 45°) influence visual attention characteristics (including number of fixations, fixation duration, total number of fixations, total fixation duration, and fixation distribution) in elite female basketball players during two‑point shooting; and (2) the relationship between these visual attention metrics and two‑point shooting accuracy.

We hypothesize that: (a) High‑intensity exercise will lead to increased fixation dispersion and reduced fixation stability across all shooting positions; (b) The 90° position will yield the highest fixation stability and shooting accuracy, while the left 45° position will result in the poorest performance for right‑handed players due to visual‑field and body‑orientation constraints; (c) Fixation duration will positively correlate with shooting accuracy, whereas excessive number of fixations may negatively correlate with accuracy, reflecting potentially reduced information‑processing efficiency [15,16].

The study holds significant theoretical and practical implications. Theoretically, by integrating Dynamic Systems Theory and Attentional Control Theory, it unveils the adaptive mechanisms of visuomotor coordination under multi‑constraint conditions, deepening our understanding of elite athletes’ cognitive advantages. Practically, the findings can inform the design of position‑specific and fatigue‑resistant visual attention training programs and may offer oculometric biomarkers for talent identification in youth athletes [17].

## Methods

### Participants

A priori power analysis was performed using G*Power software (Version 3.1.9.7) [18]. Based on a two‑way repeated‑measures ANOVA, with an assumed medium‑sized interaction effect (f = 0.25, α = 0.05, power (1 − β) = 0.80, and a correlation of 0.5 among repeated measures), the analysis indicated that a total sample size of 20 participants would be required. This effect size is consistent with those reported in previous eye‑tracking studies involving motor tasks [19,20]. To account for potential data loss or attrition, 22 participants were recruited beforehand, with two serving as backups. All participants were selected from the Beijing Normal University women’s basketball team, which had won the women’s basketball championship at the 2023 and 2025 World University Summer Games. Participants’ characteristics were as follows (mean ± SD): age 21.2 ± 1.5 years, height 178.3 ± 5.2 cm, weight 68.7 ± 4.8 kg. Each participant had over 10 years of formal training and achieved a two‑point field‑goal accuracy of at least 60% from left 45°, front 90°, and right 45° positions. All participants were right‑handed and had normal or corrected‑to‑normal vision. The study procedures adhered to the principles of the Declaration of Helsinki. The protocol was approved by the Institutional Review Board of the School of Sports and Exercise Science at Beijing Normal University (IRB‑20250626), and written informed consent was obtained from all participants.

### Apparatus

The experimental setup included a Tobii Glasses 3 portable eye tracker (Tobii, Sweden), which recorded fixation data at a sampling rate of 100 Hz. The system comprised wearable eye-tracking glasses, a recording unit, and a laptop running Tobii Pro Glasses 3 control software and Tobii Pro Lab analysis software. The design of the eye tracker minimizes obstruction to the participant’s field of view and allows extensive freedom of head and body movement while maintaining data quality, thereby supporting the collection of natural and authentic eye movement behavior [13]. Exercise intensity was monitored using the Polar Team Pro system. The system’s main station synchronized data from individual sensors with the Polar Team Pro application. The sensors captured detailed performance metrics, which were transmitted in real time via Bluetooth Smart to an iPad, enabling live monitoring throughout the training sessions [13].

### Definition of Exercise Intensity Levels

Exercise intensity levels were individually prescribed for each participant based on percentages of their maximum heart rate (HRmax), a well‑validated method for quantifying exertion levels during physical activity [19–21]. This approach ensures that the physiological demand of each condition was relative to each athlete’s personal cardiovascular capacity. First, each participant’s age‑predicted maximum heart rate was estimated using the formula: HRmax = 208 − (0.7 × age) [22]. The three discrete exercise‑intensity conditions were then defined as follows [19,23]:

- Low Intensity: 50–60% of HRmax
- Moderate Intensity: 70–80% of HRmax
- High Intensity: 85–95% of HRmax

During the experiment, real‑time heart rate was monitored using the Polar Team Pro system. The exercise protocol for each intensity block (a standardized combination of shuttle runs, defensive slides, and high‑knee drills) was actively adjusted to ensure that each participant’s heart rate was elicited and maintained within the corresponding target zone for at least 3 minutes before the commencement of shooting trials. This procedure guaranteed a consistent and standardized physiological state for all participants under each condition [23].

### Experimental scenarios

**Fig 1:**
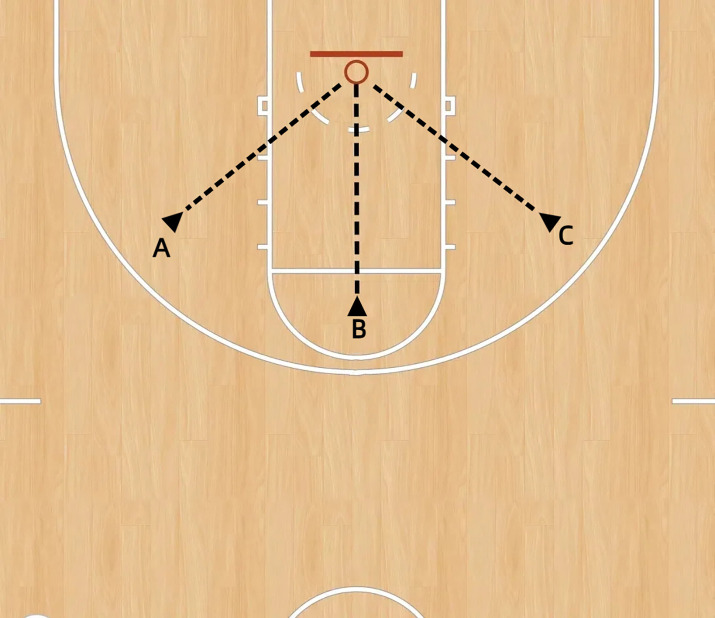
Participant’s Shot Position. **Note:** A: Shooting position from the left 45° position; B: Shooting position from the 90° (central) position; C: Shooting position from the right 45° position.

The experiment was conducted at the Beijing Normal University Gymnasium, utilizing a basketball court compliant with the official regulations of the International Basketball Federation (FIBA). Throughout the experiment, participants wore wearable eye‑tracking glasses equipped with a data‑recording unit, as well as a Polar Team heart‑rate sensor. Each participant performed shot attempts from three predetermined positions located 5 meters from the hoop: left 45° (Position A), 90° (Position B), and right 45° (Position C), as illustrated in Fig 1. Environmental conditions within the venue—including lighting, sound, and air quality—were maintained within optimal ranges and actively monitored. No external disturbances occurred during the experimental sessions.

### Experimental design

A within‑subjects repeated‑measures design with a 3 (exercise intensity: low, moderate, high) × 3 (shooting position: left 45°, center 90°, right 45°) full‑factorial design was employed to investigate their effects on two‑point shooting performance and visual‑tracking behavior. The primary dependent variables were shooting accuracy (percentage of successful shots) and eye‑movement parameters (number of fixations, fixation duration, total number of fixations, total fixation duration, and fixation distribution). The experiment was conducted from June 28 to July 2, 2025, under the supervision of the experimenter. Prior to data collection, all participants attended an introductory session detailing the experimental protocol. The experimental procedure for each participant was conducted in a single session, structured into the following phases:

1. **Preparation and Baseline Measurement:** Upon completing the informed‑consent process, participants were equipped with the Tobii Glasses 3 eye tracker and the Polar Team Pro heart‑rate monitoring system. A standardized warm‑up protocol was then administered to prepare them for the subsequent tasks.
2. **Exercise‑Intensity Intervention and Shooting Trials**: Participants underwent three distinct exercise‑intensity conditions, each followed immediately by a block of shooting trials. The sequence of the three intensity conditions was counterbalanced across all participants using a Latin‑square design to mitigate order and carryover effects. Within each intensity condition, a standardized exercise protocol (shuttle runs, defensive slides, high‑knee drills) was applied to elevate and stabilize the participant’s heart rate within a predefined target zone. Once the target intensity was confirmed and stabilized, the participant performed a series of two‑point shots from a distance of 5 meters. From each of the three predetermined positions (Position A: left 45°, B: center 90°, C: right 45°), the participant attempted 10 shots, resulting in 30 shots per intensity block. The order of these positions was randomized for each participant within each block to prevent sequential bias.
3. **Recovery Period**: A sufficient recovery interval was enforced between consecutive intensity blocks. The subsequent block commenced only after the participant’s heart rate had returned to within 10% of their pre‑exercise baseline level, ensuring that each trial started from a comparable physiological state.

This design yielded 90 total shooting trials per participant (3 intensities × 3 positions × 10 shots). The two‑point shooting accuracy for each condition was subsequently calculated as the percentage of successful shots made out of the total attempts.

### Areas of interest (AOI)

**Fig 2:**
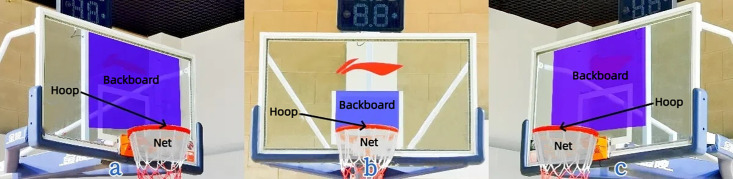
AOI based on 2-point shot fixation position. Note: a: AOI for shooting from the left 45° position; b: AOI for shooting from the 90° position; c: AOI for shooting from the right 45° position.

The AOI refers to the specific area that participants focus on during information search [24]. To establish AOIs for this study, consultations were conducted with basketball experts and instrument‑manufacturer professionals, taking into account the participants’ fixation areas during the two‑point shot (see Fig 2). Three AOIs were defined: hoop, backboard, and net [25].

### Data analysis

All eye‑tracking data were processed and analyzed using the Analysis module within Tobii Pro Lab. Only data meeting the minimum sampling‑rate criterion of 85% were included in the final analysis. All statistical analyses were performed using SPSS (Version 26) and R (Version 4.3). The significance level (α) was set at 0.05 for all tests.

To examine the effects of exercise intensity and shooting position on visual attention, a two‑way repeated‑measures analysis of variance (RM‑ANOVA) was conducted for each eye‑movement metric. The within‑ subjects factors were “Exercise Intensity” (three levels: low, moderate, high) and “Shooting Position” (three levels: left 45°, 90°, right 45°). The dependent variables included number of fixations (the number of discrete fixations per trial on a given AOI), mean fixation duration (the average length of individual fixations on a given AOI), total number of fixations (sum of fixations across all three AOIs within a trial), and total fixation duration (sum of all fixation durations within a trial). Greenhouse–Geisser corrections were applied when sphericity was violated. If a significant main or interaction effect was detected, post‑hoc comparisons were performed using Fisher’s Least Significant Difference (LSD) correction. Fisher’s LSD was chosen because it is appropriate for planned comparisons in a fully repeated‑measures design and provides adequate power while controlling Type I error in this context [26]. Effect sizes (partial eta‑squared, ηp2) are reported with 95% confidence intervals.

For RM‑ANOVA, number of fixations was analyzed as a continuous variable to assess mean differences across conditions, consistent with prior eye‑tracking studies. For generalized linear mixed‑effects models (GLMMs), number of fixations was treated as an ordinal predictor (0, 1, 2, ≥ 3) to better account for its discrete distribution at the trial level.

Given the repeated‑measures structure of the data (multiple trials per participant), the relationships between visual‑attention metrics and shooting accuracy were examined using generalized linear mixed‑effects models (GLMMs) with a binomial distribution and a logit link function, including participant‑level random intercepts. Shooting accuracy was modeled as trial‑level binary outcome (hit/miss). Separate models were fitted for each AOI (hoop, backboard, net) and each intensity condition, with shooting accuracy as the outcome and fixation metric (number of fixations or fixation duration) as the predictor. Number of fixations was treated as an ordinal predictor (0, 1, 2, ≥ 3) to better capture its discrete nature. Model assumptions were checked via residual plots. Pearson correlation coefficients are also reported for descriptive purposes, but the GLMM results are considered primary. The raw data, processed underlying dataset, and supplemental files are available as S1 File and S2 File, respectively.

## Results

### Participant characteristics and descriptive data

Table 1 presents participants’ demographic and sport‑related characteristics, as well as the means and standard deviations of the main visual‑attention variables (number of fixations and fixation duration) across AOIs, stratified by exercise intensity and shooting position.

**Table 1:** Participant characteristics and descriptive statistics of visual‑attention variables.

Variable	Mean ± SD or %
Age (years)	21.2 ± 1.5
Height (cm)	178.3 ± 5.2
Weight (kg)	68.7 ± 4.8
Training experience (years)	12.4 ± 2.1
**Two-point shooting accuracy (%)**	
Low intensity	68.06 ± 5.81 (left 45°), 71.93 ± 5.34 (90°), 69.30 ± 6.82 (right 45°)
Moderate intensity	72.46 ± 5.81 (left 45°), 76.12 ± 4.35 (90°), 73.22 ± 5.16 (right 45°)
High intensity	47.63 ± 6.58 (left 45°), 48.53 ± 6.51 (90°), 47.91 ± 6.84 (right 45°)
**Number of fixations (mean per trial)**	
Low intensity	1.35 ± 0.49 (hoop), 1.75 ± 0.55 (backboard), 0.95 ± 0.55 (net)
Moderate intensity	1.20 ± 0.41 (hoop), 1.45 ± 0.51 (backboard), 0.70 ± 0.73 (net)
High intensity	1.70 ± 0.53 (hoop), 2.05 ± 0.76 (backboard), 1.70 ± 0.47 (net)
**Fixation duration (ms, mean per trial)**	
Low intensity	662 ± 172 (hoop), 884 ± 106 (backboard), 211 ± 184 (net)
Moderate intensity	573 ± 128 (hoop), 945 ± 157 (backboard), 167 ± 173 (net)
High intensity	607 ± 144 (hoop), 790 ± 97 (backboard), 490 ± 155 (net)

### Number of fixations and fixation duration by AOI

The RM‑ANOVA results for number of fixations and fixation duration on each AOI are summarized in Tables 2 and 3, respectively. Tables are now organized into three subsections (hoop, backboard, net) with all statistical outputs clearly aligned. Tables are now organized into three subsections (hoop, backboard, net) for clarity.

**Table 2:** Number of fixations by AOI, exercise intensity, and shooting position (unit: times).

AOI	Shooting Position	Low intensity (M ± SD)	Moderate intensity (M ± SD)	High intensity (M ± SD)	Repeated measures *F* test			
					*F*	*p*	$\overset{2}{\underset{p}{\eta }}$	95% CI
**Hoop**	left 45°	1.35 ± 0.49	1.20 ± 0.41	1.70 ± 0.53				
	90°	1.85 ± 0.37	1.55 ± 0.51	2.20 ± 0.77				
	right 45°	1.45 ± 0.51	1.30 ± 0.47	1.80 ± 0.62				
**Main Effect: Intensity**					8.23	0.001	0.30	0.10, 0.48
**Main Effect: Position**					4.56	0.017	0.19	0.01, 0.35
**Interaction: Intensity × Position**					1.12	0.354	0.06	
**Backboard**	left 45°	1.75 ± 0.55	1.45 ± 0.51	2.05 ± 0.76				
	90°	1.45 ± 0.51	1.15 ± 0.37	1.65 ± 0.59				
	right 45°	1.90 ± 0.45	1.65 ± 0.49	2.20 ± 0.70				
**Main Effect: Intensity**					6.75	0.003	0.26	0.06, 0.43
**Main Effect: Position**					5.89	0.006	0.24	0.04, 0.41
**Interaction: Intensity × Position**					0.93	0.451	0.05	
**Net**	left 45°	0.95 ± 0.55	0.70 ± 0.73	1.70 ± 0.47				
	90°	0.85 ± 0.75	0.55 ± 0.51	1.50 ± 0.51				
	right 45°	0.95 ± 0.89	0.58 ± 0.51	1.45 ± 0.60				
**Main Effect: Intensity**					15.47	*p* < 0.001	0.45	0.22, 0.60
**Main Effect: Position**					2.01	0.148	0.10	
**Interaction: Intensity × Position**					1.34	0.262	0.07	

Note. M = Mean, SD = Standard Deviation. CI = Confidence Interval.

**Table 3:** Fixation duration by AOI, exercise intensity, and shooting position (unit: ms).

AOI	Shooting Position	Low intensity (M ± SD)	Moderate intensity (M ± SD)	High intensity (M ± SD)	Repeated measures *F* test			
					*F*	*p*	$\overset{2}{\underset{p}{\eta }}$	95% CI
**Hoop**	left 45°	662 ± 172	573 ± 128	607 ± 144				
	90°	978 ± 153	988 ± 155	808 ± 101				
	right 45°	691 ± 124	649 ± 125	671 ± 129				
**Main Effect: Intensity**					12.34	*p <* 0.001	0.39	0.16, 0.55
**Main Effect: Position**					6.72	0.003	0.26	0.06, 0.43
**Interaction: Intensity × Position**					1.58	0.184	0.08	
**Backboard**	left 45°	884 ± 106	945 ± 157	790 ± 97				
	90°	619 ± 169	463 ± 162	594 ± 146				
	right 45°	861 ± 94	891 ± 137	743 ± 115				
**Main Effect: Intensity**					8.93	*p* < 0.001	*0.32*	0.10, 0.49
**Main Effect: Position**					4.25	0.021	0.18	0.01, 0.35
**Interaction: Intensity × Position**					1.12	0.352	0.06	
**Net**	left 45°	211 ± 184	167 ± 173	490 ± 155				
	90°	211 ± 184	167 ± 173	498 ± 166				
	right 45°	202 ± 171	153 ± 153	431 ± 113				
**Main Effect: Intensity**					32.15	*p* < 0.001	0.63	0.42, 0.74
**Main Effect: Position**					1.89	0.167	0.09	
**Interaction: Intensity × Position**					0.94	0.445	0.05	

Note. M = Mean, SD = Standard Deviation. CI = Confidence Interval.

Post‑hoc tests indicated that number of fixations under high intensity were significantly greater than under low and moderate intensities for all AOIs (*p* < 0.01), following the pattern: high > low > moderate. For the hoop AOI, the 90° position elicited the highest number of fixations (*p* < 0.05). For the backboard AOI, the right 45° position elicited the highest number of fixations (*p* < 0.05) longest durations again at the right 45° position. No significant interactions were found (*p* > 0.05).

Post‑hoc analyses showed a clear hierarchy for fixation duration: high > moderate > low intensity (*p* < 0.01). For the hoop AOI, the 90° position attracted the longest fixation durations (*p* < 0.05). For the backboard AOI, the left 45° position showed the longest durations (*p* < 0.05).

### Total number of fixations and total fixation duration

The analysis of global visual metrics (refer to Table 4, 5) showed a significant main effect of exercise intensity on both total number of fixations (*F*_(2,38)_ = 24.57, *p* < 0.001, $\overset{2}{\underset{p}{\eta }}$= 0.56, 95% CI [0.34, 0.68]) and total fixation duration (*F*_(2,38)_ = 18.73, *p* < 0.001, $\overset{2}{\underset{p}{\eta }}$= 0.50, 95% CI [0.27, 0.64]). For both measures, values were highest under high intensity, followed by low and then moderate intensity (all pairwise comparisons *p* < 0.01). A significant main effect of shooting position was found for total number of fixations (*F*_(2,38)_ = 6.89, *p* = 0.003, $\overset{2}{\underset{p}{\eta }}$= 0.27, 95% CI [0.06, 0.44]), with the right 45° position resulting in the highest number of fixations. No other main or interaction effects were significant for total fixation duration.

**Table 4:** Total Number of Fixations during Two-Point Shooting by Exercise Intensity and Position (Unit: Times).

Shooting Position	Low intensity (M ± SD)	Moderate intensity (M ± SD)	High intensity (M ± SD)	Repeated measures *F* test			
				*F*	*p*	$\overset{2}{\underset{p}{\eta }}$	95% CI
Left 45°	4.05 ± 1.00	3.35 ± 1.14	5.45 ± 1.05				
90°	4.15 ± 1.02	3.25 ± 0.85	5.35 ± 1.18				
Right 45°	4.30 ± 1.17	3.50 ± 0.89	5.55 ± 0.89				
Main Effect: Intensity				24.57	*p* < 0.001	0.56	0.34, 0.68
Main Effect: Position				6.89	0.003	0.27	0.06, 0.44
Interaction: Intensity × Position				1.34	0.262	0.07	

Note. M = Mean, SD = Standard Deviation. CI = Confidence Interval.

**Table 5:** Total Fixation Duration during Two-Point Shooting by Exercise Intensity and Position (Unit: Ms).

Shooting position	Low intensity (M ± SD)	Moderate intensity (M ± SD)	High intensity (M ± SD)	Repeated measures *F* test			
				*F*	*p*	$\overset{2}{\underset{p}{\eta }}$	95% CI
Left 45°	1757 ± 101	1649 ± 163	1887 ± 74				
90°	1808 ± 73	1618 ± 123	1900 ± 102				
Right 45°	1755 ± 111	1685 ± 141	1844 ± 89				
Main Effect: Intensity				18.73	*p* < 0.001	0.50	0.27, 0.64
Main Effect: Position				2.14	0.131	0.10	
Interaction: Intensity × Position				1.56	0.194	0.08	

Note. M = Mean, SD = Standard Deviation. CI = Confidence Interval.

### Fixation distribution

**Fig 3:**
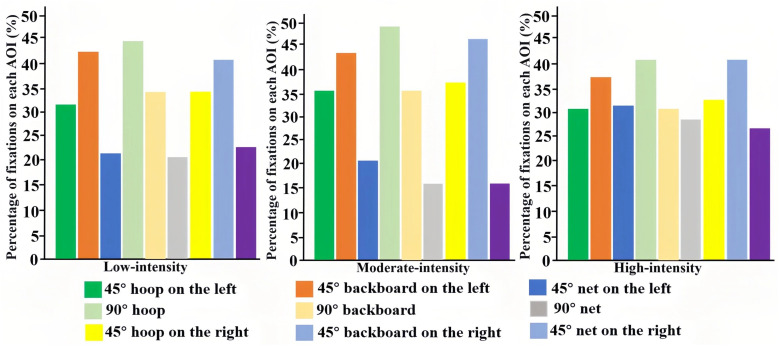
Percentage distribution of the number of fixations across AOIs by exercise intensity and shooting position.

**Fig 4:**
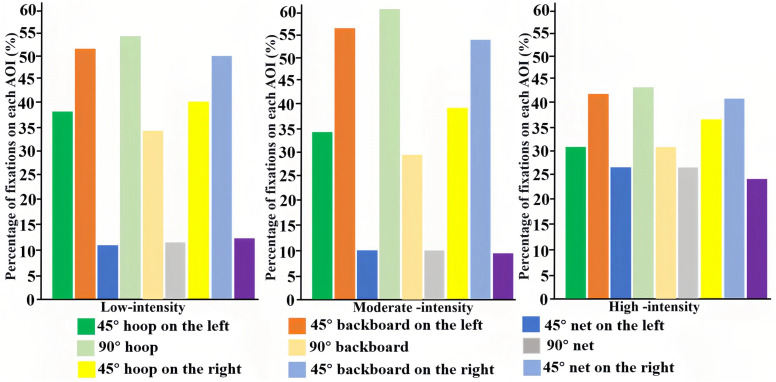
Percentage distribution of fixation duration across AOIs by exercise intensity and shooting position.

The distribution of visual attention across AOIs varied systematically with shooting position and exercise intensity. Figs 3 and 4 display the percentage distribution (rather than absolute values) of number of fixations and fixation duration, respectively, to directly support the proportional descriptions in the text.

At the 90° position, the hoop received the highest proportion of both number of fixations (45–48%) and fixation duration (43–61%) across all intensity levels, indicating it was the primary attentional focus. As intensity increased from low to high, the proportion of attention allocated to the net increased substantially for number of fixations (20% to 28%) and fixation duration (12% to 26%), while the share for the hoop and backboard decreased.

For shots from the left and right 45° positions, the backboard was the dominant AOI, attracting the highest proportion of number of fixations (38–47%) and fixation duration (41–57%). A similar effect of intensity was observed: the net attracted a greater share of number of fixations (increasing to 26–31%) and fixation duration (increasing to 23–26%) under high intensity, at the expense of the backboard and hoop. Overall, visual attention was most stable and concentrated during moderate‑intensity trials, which were associated with the lowest total number of fixations and fixation duration.

### Relationship between fixation metrics and shooting accuracy

**Fig 5:**
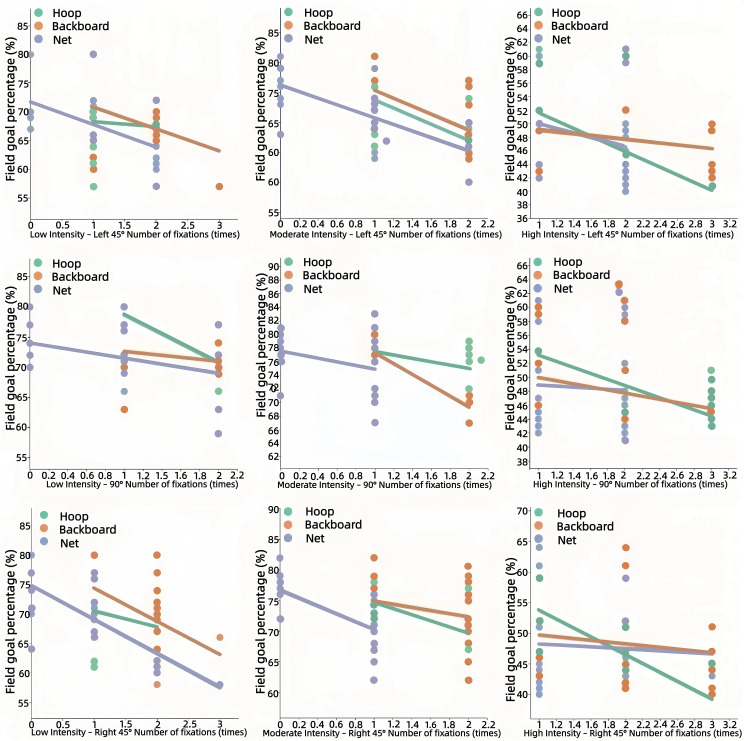
Relationship Between the Number of Fixations and Shooting Accuracy.

Generalized linear mixed‑effects models (GLMMs) with a binomial distribution (controlling for participant‑level random effects) revealed the following patterns:

Number of fixations: Under moderate intensity, a higher number of fixations on the hoop was associated with lower odds of a successful shot (*β* = −0.542, *p* = 0.013). No significant relationships were found at low or high intensity (refer to Fig 5).

**Fig 6:**
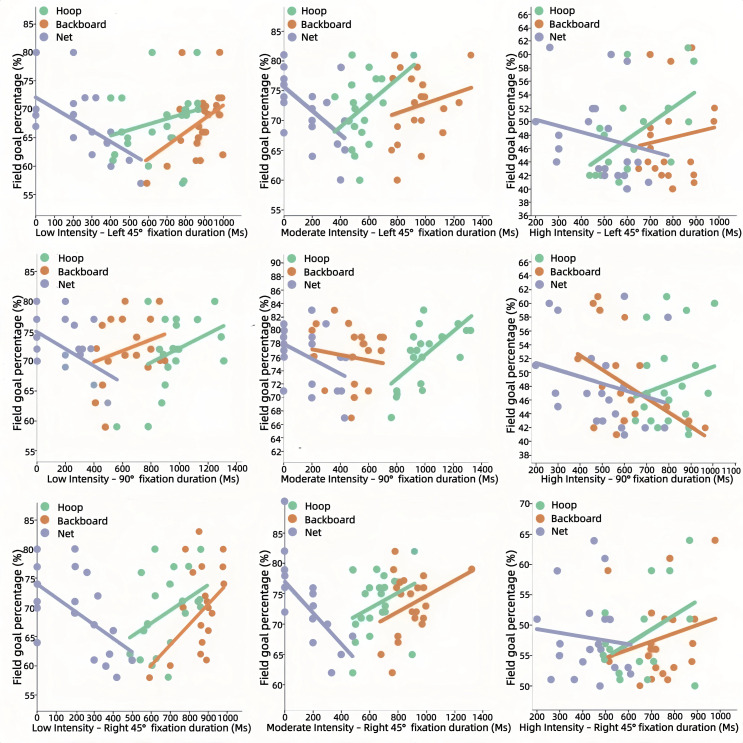
Relationship Between Fixation Duration and Shooting Accuracy.

Fixation duration: Under high intensity, longer fixation duration on the hoop was associated with higher odds of a successful shot (*β* = 0.499, *p* = 0.021). No significant relationships were observed at low or moderate intensity (refer to Fig 6).

## Discussion

The present study investigated how exercise intensity and shooting position influence fixation behavior and 2‑point shooting accuracy in elite female basketball players. The findings largely support our hypotheses and reveal systematic adaptations in visual attention under different task constraints.

### Spatial fixation metrics: Number of fixations

As hypothesized, high‑intensity exercise led to a significant increase in number of fixations across all AOIs, indicating elevated cognitive load and less efficient visual search. This aligns with attentional control theory, which posits that high physiological demand impairs top‑down cognitive control, leading to more exploratory fixation behavior [27,25]. In contrast, the lowest total number of fixations and the highest proportion of fixations dedicated to the hoop during moderate intensity reflect a focused “narrow‑external” attentional focus. This efficient strategy, likely honed through long‑term training, allows athletes to filter irrelevant stimuli and direct attention to the most critical area, thereby optimizing information seeking [28]. The superior attentional focus at moderate intensity may be attributed to an optimal psychophysiological state, whereas low intensity might represent sub‑optimal arousal and high intensity degrades cognitive control through fatigue [25,29].

Contrary to our hypothesis that the left 45° position would yield the poorest performance for right‑handed players, the right 45° position actually elicited the highest number of fixations for the backboard. This may reflect visual‑field asymmetries or biomechanical adjustments specific to elite athletes (e.g., a preference for preparing shots from the right side), warranting further study [30,31].

### Temporal fixation metrics: Fixation duration

Fixation duration reflects the depth and efficiency of information processing. The shortest total fixation duration during moderate intensity indicates rapid and efficient information extraction, whereas prolonged durations under high intensity suggest increased processing difficulty and a compensatory need for extended analysis [25,32]. The hoop attracted the longest fixation durations across all intensities, confirming it as the primary information source. According to dual‑process theories of cognitive control, optimal performance depends on a balance between proactive (goal‑directed) and reactive (stimulus‑driven) control [28,33]. We posit that moderate intensity facilitates optimal proactive control, enabling efficient processing. High intensity likely disrupts this balance, depleting cognitive resources and forcing a shift toward more effortful and time‑consuming reactive control, thereby reducing processing efficiency [6,1].

### Visual attention and 2-point shooting accuracy

The GLMM results partially supported our hypotheses. The negative relationship between number of fixations and accuracy across intensities suggests that fewer, more stable fixations are characteristic of an optimal fixation pattern [34,35]. The positive relationship between fixation duration on the hoop and accuracy under high intensity implies that athletes attempt to compensate for reduced processing efficiency by prolonging information extraction, albeit with limited success as accuracy remains lower than at moderate intensity. The negative correlation between net fixation time and accuracy highlights that attention to irrelevant areas is detrimental to performance. The optimal performance at moderate intensity can be linked to an ideal arousal level that enhances cognitive function, potentially supported by beneficial neuroendocrine responses [36]. Conversely, high intensity may elevate metabolites like blood lactate and ammonia, which can impair cognitive function and motor performance [37,38].

### Limitations and future directions

Several limitations should be acknowledged. First, all participants were right‑handed elite female basketball players from a single championship team, which may limit generalizability to other populations (e.g., male athletes, other competitive levels, left‑handed players). Second, exercise intensity was defined using an age‑predicted HRmax formula rather than direct maximal testing; although this is a common field method, it may introduce some individual error. Third, while we interpret the findings through theoretical frameworks linking arousal, cognitive control, and visual search, we did not collect direct physiological measures of fatigue or arousal (e.g., lactate, cortisol). Future studies could incorporate such biomarkers to more directly test the psychophysiological mechanisms. Fourth, the study focused on two‑point shots from three positions; expanding to three‑point shots or more dynamic game‑like scenarios would further enhance ecological validity. Finally, the analysis of quiet‑eye duration was not included; future work could specifically examine QE as a function of intensity and position.

### Practical implications

The findings offer concrete guidance for coaches and sports scientists. Visual‑attention training should be tailored to specific shooting positions and integrated with intensity‑specific drills. For example, under moderate intensity, drills that promote a focused, stable fixation on the hoop are likely most beneficial. Under high intensity, training could emphasize maintaining longer fixations on the hoop despite physiological stress. Position‑specific training should account for the shift in attentional focus (e.g., more backboard‑oriented fixations at 45° angles). Such tailored approaches may help athletes maintain shooting accuracy under the varied demands of actual competition.

## Conclusions

Exercise intensity systematically alters visual attention. Moderate intensity fosters an optimal state for deploying focused attention and efficient cognitive processing, while high intensity imposes a significant cognitive load that disrupts elite‑athlete fixation patterns and impairs performance. Shooting position also modulates visual strategy, with the hoop being the primary focus at 90° and the backboard gaining importance at 45° angles. These findings underscore the value of integrating intensity‑ and position‑based visual training into basketball practice to enhance shooting performance under realistic game conditions.

## Supporting information

S1 File Underlying dataset (English version). Processed dataset used for statistical analysis, with English variable labels.
(XLSX)

S2 File Supplemental files. Contains supplementary materials including eye-tracking calibration logs and task protocol details.
(DOCX)
