# Supplementary material for: Exercise intensity and shooting position modulate fixation behavior and 2-point shooting accuracy in elite female basketball players: An eye-tracking study
Source: PLoS One. 2026 Apr 30;21(4):e0348017. doi: 10.1371/journal.pone.0348017 (PMC13132191; doi:10.1371/journal.pone.0348017)
Supplement: S2 File — Contains supplementary materials including eye-tracking calibration logs and task protocol details. (DOCX) [file pone.0348017.s002.docx]

**Participant informed consent**

**Participants Number**：

| **Name** |  | **Gender** |  | | **Age** | | | |  |
| --- | --- | --- | --- | --- | --- | --- | --- | --- | --- |
| **Height（cm）** |  | **Weight（kg）** |  | | **Educational attainment** | | | |  |
| **Professional** |  | **Student ID** |  | | **Class** | | | |  |
| **Years of formal training** |  | **Strong side hand** |  | | **Position on the field** | | | |  |
| **Experiment Name** | Exercise Intensity and Shooting Position Modulate Fixation Behavior and 2-Point Shooting Accuracy in Elite Female Basketba Players: An Eye-Tracking Study | | | | | | | | |
| **Identity information** | **Name/Title** |  | | **Research unit** | | |  | | |
| **Principal Investigator** |  | | | | | | | | |
| **Type of experimenter** |  | | | | | | | | |
| **Type of experimenter** |  | **Type of experimenter** |  | | | **Email：** | |  | |
| **Purpose：**  To study the visual attention characteristics of outstanding female college basketball players when shooting two-point shots under different exercise intensities and their relationship with shooting percentage.  **Procedure**  A within‑subjects repeated‑measures design with a 3 (exercise intensity: low, moderate, high) × 3 (shooting position: left 45°, center 90°, right 45°) full‑factorial design was employed to investigate their effects on two‑point shooting performance and visual‑tracking behavior. The primary dependent variables were shooting accuracy (percentage of successful shots) and eye‑movement parameters (number of fixations, fixation duration, total number of fixations, total fixation duration, and fixation distribution). The experiment was conducted from June 28 to July 2, 2025, under the supervision of the experimenter. Prior to data collection, all participants attended an introductory session detailing the experimental protocol. The experimental procedure for each participant was conducted in a single session, structured into the following phases:  1. Preparation and Baseline Measurement: Upon completing the informed‑consent process, participants were equipped with the Tobii Glasses 3 eye tracker and the Polar Team Pro heart‑rate monitoring system. A standardized warm‑up protocol was then administered to prepare them for the subsequent tasks.  2. Exercise‑Intensity Intervention and Shooting Trials: Participants underwent three distinct exercise‑intensity conditions, each followed immediately by a block of shooting trials. The sequence of the three intensity conditions was counterbalanced across all participants using a Latin‑square design to mitigate order and carryover effects. Within each intensity condition, a standardized exercise protocol (shuttle runs, defensive slides, high‑knee drills) was applied to elevate and stabilize the participant's heart rate within a predefined target zone. Once the target intensity was confirmed and stabilized, the participant performed a series of two‑point shots from a distance of 5 meters. From each of the three predetermined positions (Position A: left 45°, B: center 90°, C: right 45°), the participant attempted 10 shots, resulting in 30 shots per intensity block. The order of these positions was randomized for each participant within each block to prevent sequential bias.  3. Recovery Period: A sufficient recovery interval was enforced between consecutive intensity blocks. The subsequent block commenced only after the participant’s heart rate had returned to within 10% of their pre‑exercise baseline level, ensuring that each trial started from a comparable physiological state.  This design yielded 90 total shooting trials per participant (3 intensities × 3 positions × 10 shots). The two‑point shooting accuracy for each condition was subsequently calculated as the percentage of successful shots made out of the total attempts. | | | | | | | | | |
| **Costs：**  This study will not charge you or your medical insurance any fees. | | | | | | | | | |
| **Risks and Side effects：**  The experiment poses no personal danger and has no side effects at all. You can request to terminate the experiment at any time without any reason. | | | | | | | | | |
| **Benefits：**  You will receive a gift by participating in this study. | | | | | | | | | |
| **Confidentiality：**  The results of this study may be published in academic journals/books or used for teaching purposes. However, your name or any other information that can confirm you will not appear in any published or teaching materials unless you give your permission. In addition, any photos, audio recordings or video recordings obtained during this research that can confirm your identity will only be used after obtaining your written permission. | | | | | | | | | |
| **Subject** **Statement：**  I confirm that I have been informed of the purpose, process, possible risks and side effects, as well as potential benefits and costs of this study. All my questions have been answered satisfactorily. I have read this informed consent form of the experimenter in detail. My signature below indicates my willingness to participate in this research.  **Signature: Date:** | | | | | | | | | |
| **Experimenter** **Statement**  I have explained the purpose of the research, the research procedures, potential dangers and discomforts, as well as the rights and interests of the experimenters, and have answered the questions related to the research to the best of my ability. I have verified the age and other identity information of the experimenter.  **Signature: Date:** | | | | | | | | | |
